# Roles of TGF-**β** Signals in Endothelial-Mesenchymal Transition during Cardiac Fibrosis

**DOI:** 10.4061/2011/724080

**Published:** 2011-11-30

**Authors:** Yasuhiro Yoshimatsu, Tetsuro Watabe

**Affiliations:** Department of Molecular Pathology, Graduate School of Medicine, The University of Tokyo, Hongo, Bunkyo-ku,Tokyo 113-0033, Japan

## Abstract

Most cardiac diseases caused by inflammation are associated with fibrosis in the heart. Fibrosis is characterized by the accumulation of fibroblasts and excess deposition of extracellular matrix (ECM), which results in the distorted organ architecture and function. Recent studies revealed that cardiac fibroblasts are heterogeneous with multiple origins. Endothelial-mesenchymal transition (EndMT) plays important roles in the formation of cardiac fibroblasts during pathological settings. EndMT is regulated by signaling pathways mediated by cytokines including transforming growth factor (TGF)-**β**. Better understanding of the mechanisms of the formation of cardiac fibroblasts via EndMT may provide an opportunity to develop therapeutic strategies to cure heart diseases.

## 1. Introduction

Many of heart injuries end up in a common final pathway of pathologic tissue remodeling and fibrosis (defined by deposition of collagens, elastin, tenascin, and other matrix proteins), leading to the development of heart failure. Myocardial fibrosis induced by cardiac fibroblasts plays a dual role in cardiac remodeling after injury. While fibrosis plays important roles in wound healing, it also contributes to ventricular stiffening and heart failure progression. Recent reports have revealed that cardiac fibroblasts originate through the mesenchymal transition of endothelial cells (ECs) [1], which is termed “endothelial-mesenchymal transition (EndMT).” Here, after updating current views on the sources of cardiac fibroblasts, we will review epithelial mesenchymal transition (EMT), in which epithelial cells acquire mesenchymal phenotype, since this process has many similarities with EndMT and lays the groundwork for understanding EndMT. We will then review the roles of EndMT in physiological and pathological settings and address its potential mechanisms.

## 2. Source of Fibroblasts during Cardiac Fibrosis

Several lines of evidence suggest that cardiac fibroblasts are a heterogeneous population and derive from various distinct tissue niches in physiological and pathological conditions. During embryonic heart development, cardiac fibroblasts are differentiated from epicardium or endocardium of the heart [2–6]. In a healthy adult heart, cardiac fibroblasts reside in the interstitial tissue within the myocardium. Some reports have shown that heart-resident cardiac fibroblasts are the major source of tissue fibrosis associated with ischemic heart failure and hypertrophy [7, 8]. In addition, fibroblasts originated from bone marrow-derived cells including CD45-positive hematopoietic stem cells (HSCs) have also been shown to significantly contribute to remodeling of the injured heart [9–13]. Finally, emerging evidence suggests that a subset of cardiac fibroblasts is originated from ECs in a mouse model of pressure overload [1]. This endothelial mesenchymal transition has common features with epithelial mesenchymal transition. Taken together, cardiac fibroblasts are thought to be derived from resident fibroblasts, bone marrow-derived cells, and ECs.

## 3. Epithelial-Mesenchymal Transition (EMT)

**Figure 1:**
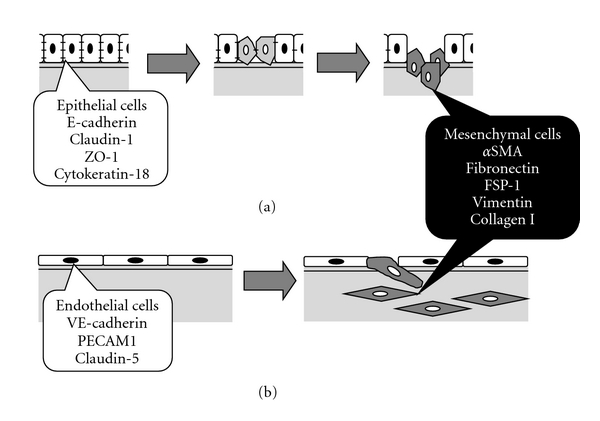
Schematic representation of mesenchymal transition of epithelial (a) and endothelial (b) cells.

EMT is a process in which epithelial cells lose their polarity and cell-to-cell contacts and undergo a dramatic remodeling of the cytoskeleton (Figure 1(a)) [14, 15]. During EMT, there is a marked decrease in the expression of epithelial markers including E-cadherin, claudin, zona occludens-1 (ZO-1), and cytokeratin-18, and concurrent increase in the expression of mesenchymal markers including smooth muscle *α*-actin (SMA), fibroblast-specific protein 1 (FSP1; also known as S100A4), fibronectin, and collagens. Furthermore, the mesenchymal cells manifest migratory and proliferative phenotypes. EMT has been implicated in many critical steps during embryonic development including gastrulation and formation of various tissues or cell clusters (neural crest, musculoskeletal system, cranial facial structures, and peripheral nervous system). 

Emerging evidence suggests that EMT is also involved in tissue injury leading to tissue fibrosis [16]. For example, EMT is associated with progressive fibrosis in kidney disease. While fibroblasts are not particularly abundant in the normal kidney, there is a marked increase in the number of fibroblasts at the onset of fibrogenesis [17]. Furthermore, EMT also contributes to the fibrotic responses observed in several lung pathologies, such as rejecting lung allografts, silica-induced lung carcinogenesis, and in idiopathic pulmonary fibrosis [18].

## 4. Signaling Pathways Mediated by TGF-*β* Family Members

Although EMT has been implicated in many pathological processes described above, our knowledge of the molecular events that govern EMT remains relatively undefined. Transforming growth factor-*β* (TGF-*β*) is a multifunctional cytokine that plays many aspects of cell development, differentiation, and homeostasis, and suppresses their uncontrolled proliferation and transformation. 

**Figure 2:**
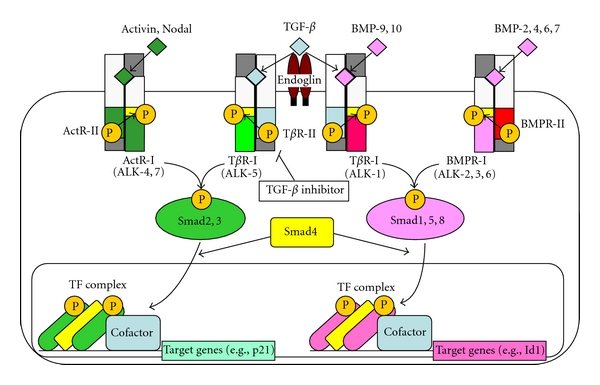
Smad signal transduction pathways mediated by TGF-*β* and BMP family members.

TGF-*β* belongs to the TGF-*β* superfamily, which includes 33 members in mammals; these include TGF-*β*s, bone morphogenetic proteins (BMPs), activins and inhibins, Nodal, myostatin, and Müllerian-inhibiting substance (MIS, also known as anti-Müllerian hormone) [19]. Members of the TGF-*β* family bind to two different types of serine/threonine kinase receptors (Figure 2). Upon ligand binding, the constitutively active type II receptor kinase phosphorylates the type I receptor which, in turn, activates the downstream signal transduction cascades, including Smad pathways. TGF-*β*s, activin, and Nodal signal through type I receptors are known as activin receptor-like kinase (ALK)-4, -5, and -7, respectively. The activated type I receptors phosphorylate receptor-regulated Smad proteins (R-Smads). Smad2 and 3 transduce signals for TGF-*β*s and activins, while Smad1, 5, and 8 are specific for signaling of BMPs [20]. As an exception, ALK-1, preferentially expressed in ECs, binds TGF-*β* and activates Smad1/5 pathways [21]. Recently, BMP-9 and BMP-10 were reported to bind to ALK-1 [22, 23]. Once activated, R-Smads complex with the common mediator Smad4 (co-Smad) and translocate to the nucleus, where Smad complexes regulate transcription of target genes through their interaction with various transcription factors. 

**Figure 3:**
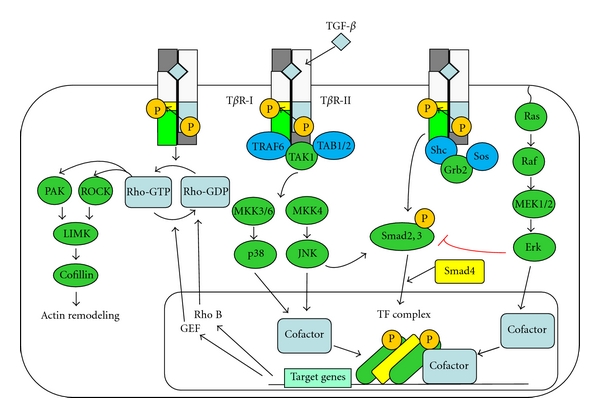
Non-Smad signal transduction pathways mediated by TGF-*β* family members.

In addition, TGF-*β* has been shown to activate diverse non-Smad parallel downstream pathways, such as extracellular signal-regulated kinase (ERK), c-Jun NH2-terminal kinase (JNK), and p38 MAP kinase [24] (Figure 3). Furthermore, TGF-*β* activates the RhoA during EMT [25]. The Rho family of small GTPases is comprised of RhoA, Rac1, and Cdc42 and regulate the formation of stress fibers, lamellipodia, and filopodia, respectively [26]. Moreover, RhoA activation is also essential for TGF-*β* stimulation of SMA expression in renal epithelial cells undergoing EMT [27]. Taken together, these studies point to the overall importance of noncanonical TGF-*β* signaling as well as canonical TGF-*β*/Smad signaling to induce EMT in epithelial cells.

## 5. Other Signaling Pathways and Transcription Factors Involved in EMT

Several transcription factors, such as Snail, Slug, *δ*EF1, SIP1, and Twist, have been implicated in EMT [28]. Snail, a zinc-finger containing transcription factor, represses E-cadherin expression and induces EMT when overexpressed in epithelial cells [29, 30]. Knockout mice deficient for Snail gene die at gastrulation as they fail to undergo a complete EMT process, forming an abnormal mesodermal layer that maintains E-cadherin expression [31]. While TGF-*β* signals have been shown to induce the expression of Snail, SIP1, and *δ*EF1 during EMT of mammary epithelial cells [32, 33], the causal relationship between TGF-*β*-induced Snail expression and EMT has not yet been fully elucidated. In inflammatory conditions in which fibrosis is accelerated, the effect of inflammatory cytokines such as TNF-*α* cannot be disregarded. Stability of Snail protein was found to be increased by TNF-*α* in most cancer cell lines, which results in the elevated migration and invasion [34]. TNF-*α* also imparts breast cancer cells with a stem cell-like phenotype by inducing Slug expression via NF*κ*B [35], a major transcription factor that functions downstream of TNF-*α*. Furthermore, several lines of evidence suggest that TGF-*β* and TNF-*α* synergistically induce the EMT of lung carcinoma cells [36, 37], suggesting that TGF-*β* and other inflammatory signals collaborate to induce the mesenchymal transition of epithelial cells via regulation of EMT-related transcription factors. It is of our great interest that EMT-related transcription factors may also be involved in EndMT. Therefore, we will later discuss recent works on the involvement of EMT-related transcription factors in EndMT.

## 6. Endothelial-Mesenchymal Transition (EndMT)

Blood vessels are lined by ECs and, except for capillaries, surrounded by mural cells (pericytes or smooth muscle cells). ECs exhibit a wide range of phenotypic variability depending on local physiological requirement throughout the vascular network [38]. Furthermore, in pathologic conditions, the endothelium can be affected in a number of ways. One of the most remarkable ways is an extreme form of endothelial plasticity known as EndMT. During EndMT, resident ECs delaminate from a polarized cell layer and invade the underlying tissue (Figure 1(b)). This so-called mesenchymal phenotype can be characterised by loss of cell-cell junctions, acquisition of invasive and migratory properties, loss of EC markers, such as VE-cadherin and platelet endothelial cell adhesion molecule-1 (PECAM-1, also known as CD31), and gain of mesenchymal markers, such as SMA and FSP1 [1, 39–43]. Several lines of evidence have suggested that EndMT is involved not only in pathological [1, 43–45] but also in physiological conditions, such as the development of heart [46].

## 7. EndMT during Heart Development

During heart development, heart tube consists of two layers, an inner endocardium and an outer myocardium, which are separated by an acellular layer of extracellular matrix, so-called the cardiac jelly. Endocardial cells acquire endothelial cell markers, such as VE-cadherin and PECAM1. After the formation of cardiac cushion, signals from the outflow tract and atrioventricular (AV) myocardium stimulate transformation of the endocardial cells around the AV cushion and outflow tract into mesenchymal cells through EndMT to generate the primordia of the valves and membranous septa [47, 48]. This notion that valvular fibroblasts in the heart originate from endocardium was clearly confirmed by lineage analysis. In order to study cell fates in mice, Zeisberg and colleagues made use of the *R26Rosa-lox-STOP-lox-LacZ; Tie2-Cre* (*Rosa26-Tie2*) double-transgenic reporter mice [1]. In *Rosa26-Tie2* mice, EC-specific Cre recombinase removes the floxed STOP cassette, and the LacZ reporter gene is constitutively expressed under the control of ubiquitous R26R promoter. Therefore, all endothelial cells and their derivatives in this transgenic mouse will be irreversibly labeled with LacZ (*β*-galactosidase). Through this fate mapping studies, Zeisberg and colleagues found that EndMT occurs only in heart valves in physiological condition.

Slug, a well-known TGF-*β* target gene, especially, in the context of EMT, is a direct target of Notch and is required for initiation of cardiac EndMT [46]. Slug (−/−) embryos had significant fewer mesenchymal cells than wild-type control, indicating Slug is required for cardiac EndMT. Slug, but not Snail, is directly upregulated by Notch in ECs and is required for Notch-mediated repression of VE-cadherin expression. They also observed that CD31 expression is downregulated in ECs overexpressing Notch or Slug. These results suggest that Slug is involved in repressing EC markers although upregulation of mesenchymal markers depends on other transcription factors. Slug may be involved in only repressing EC markers during EndMT. Slug-deficient embryos increased Snail expression and knockdown of Snail expression in Slug (−/−) embryos significantly reduced AV canals, indicating that Snail expression can compensate for Slug deficiency and the redundant role of these Snail family members for cardiac cushion formation during cardiac EndMT.

## 8. Roles of TGF-*β* Family Signals in the EndMT during Heart Development

*In vitro* studies using tissue explants cultures in three-dimensional collagen gels have revealed the roles of TGF-*β* family members in cushion formation. More recently, analyses of knockout mice deficient in various signaling components have also shown that in addition to TGF-*β* family signals, multiple signaling pathways are involved in directing EndMT during cushion formation [49, 50]. 

Both *in vitro* and *in vivo* studies have shown that TGF-*β*2 plays central roles in endocardial EndMT. Among three members of TGF-*β* family (TGF-*β*1, 2, and 3), only TGF-*β*2 is expressed and required for endocardial cushion cell transformation in the mouse [51] since TGF-*β*2-deficient mice have multiple defects in the formation of AV cushion [52]. However, in the absence of endoglin, TGF-*β*2 was not able to induce AV cushion transformation, suggesting that this coreceptor plays an essential role in EndMT. Additionally, loss of the ALK-5, a type I TGF-*β* receptor, or the coreceptor endoglin decreased the number of mesenchymal cells in AV explant cultures [53]. To our interest, although endothelial-specific deletion of the TGF-*β* type II receptor (T*β*RII) prevented EMT in *in vitro* cultures, there were no EMT defects observed in the developing mouse embryo [54], suggesting a compensatory mechanism by other members of the TGF-*β* superfamily. 

We previously reported that TGF-*β* plays important roles during mesenchymal differentiation of mouse embryonic stem cell-derived endothelial cells (MESECs). TGF-*β*2 induced the differentiation of MESECs into mural cells with decrease in expression of an endothelial marker, claudin-5, and increase in that of mural markers, SMA, SM22*α*, and calponin [55]. We also showed that TGF-*β*-induced Snail expression is necessary for the EndMT of MESEC, suggesting that EndMT and EMT share a common signal-transcription network to induce the changes in the expression of endothelial/epithelial and mesenchymal markers. 

Multiple reports have shown that BMP2 released from the myocardium acts as an inductive signal initiating the onset of EndMT [56, 57]. This myocardial signal leads to increased TGF-*β* synthesis in the endocardial cells, and this autocrine TGF-*β* induces EndMT [40]. While BMP signals induce EndMT in explant cultures [56], only limited genetic evidence has been obtained due to the early lethality of mice deficient in several BMP components. BMPs signal through specific type I receptors including ALK-2, 3, and 6. The ALK-2 deficiency in the endothelium resulted in the defective AV canal formation [58], and endothelial-specific deletion of ALK-3 [59] showed that ALK-3 is also required for EndMT of the heart. Additionally, cardiac muscle lacking ALK-3 expression is also deficient in TGF-*β*2 [60], supporting a two-step model for EndMT. Analyzing double-mutant embryos of the homeobox transcription factors Msx1 and Msx2, downstream effectors, and upstream regulators of BMP signaling revealed reduced number of SMA-positive cells in the AV cushions [61]. Smad4, a common downstream mediator of both TGF-*β* and BMP signals, is crucial for heart development. Smad4-deficient endocardial cells fail to proliferate and do not undergo EndMT, retaining morphological features of ventricular endocardium and decreased Id2 gene expression. Smad4 and GATA-4 interact in endocardium to induce Id2 expression cooperatively. In accordance with these findings, human GATA-4 mutations by which GATA-4 cannot bind to Smad4 cause atrioventricular septal defects due to loss of Id2 expression [62]. Taken together, TGF-*β* and BMP signals play essential roles in the EndMT-mediated formation of the valves and septa of the developing heart, providing a source of mesenchymal cells to form the valves and septa.

## 9. Roles of EndMT in Cardiac Fibrosis

Using the Rosa26-Tie2 reporter mouse described above, Zeisberg and colleagues performed fate mapping to trace the origin of the fibroblasts in cardiac fibrosis. Cardiac fibrosis was induced in these mice by exposing the heart to pressure overload for 5 days via aortic banding. Analysis of the fibrotic lesions revealed the presence of cells expressing both *β*-galactosidase (LacZ) and FSP1, a fibroblast marker. Analyzing fibrotic hearts of transgenic mice expressing green fluorescent protein (GFP) under the control of the FSP1 promoter showed the presence of GFP and PECAM-1 double-positive cells. These studies demonstrated that endothelial cells undergo EndMT and contributed to the total pool of cardiac fibroblasts, as observed during formation of the AV cushion in developing heart. Interestingly, in the fibrotic heart, approximately one-third of all fibroblasts were found to be originated from endothelial cells. 

Two other groups have described the roles of EndMT in cardiac fibrosis. They used coimmunostaining method of endothelial and mesenchymal markers, instead of the irreversible fate mapping method to identify EndMT. Widyantoro and colleagues showed that endothelial cell-derived Endothelin-1(ET-1) promotes cardiac fibrosis via EndMT in streptozotocin-induced diabetic mouse model [44]. Cardiac function is decreased and perivascular fibrosis is enhanced in diabetic mice. Diabetes upregulated the production of ET-1 and TGF-*β*. Fibrosis markers, S100A4/FSP-1, Vimentin, and collagen 1*α* are regulated in CD31-positive cells, and an EC marker, VE-cadherin, is downregulated in diabetic mice. EC-specific deletion of ET-1 ameliorates these EndMT-related gene expression changes. They also addressed the mechanism by which ET-1 induces EndMT in cultured EC. They showed that ET-1 induces the TGF-*β* expression and claimed that TGF-*β*-Akt-Snail-axis is involved in this EndMT.

Ghosh and colleagues found deletion of plasminogen activator inhibitor-1 (PAI-1) results in constitutive active TGF-*β* signaling in aged mice [45]. Expression of Collagen1A1, TGF-*β*2, MMP-2, FGF-2, and its receptor, FGFR2, is also upregulated in aged mice. Importantly, in resemblance to EndMT, FGF signals play important roles in producing activated fibroblasts during EMT [63, 64]. The number of Mac-3-positive cells increased in the PAI-1 null heart, indicating that inflammation occurred and infiltrating macrophage may contribute to the pathogenesis and progression of cardiac fibrosis in PAI-1-deficient mice. Phosphorylation of Smad2 and ERK1/2 is also enhanced in the PAI-1 null heart, indicating upregulation of TGF-*β* signals. In their *in vitro* analysis, expression of CD31 decreases in concomitant with upregulation of FSP-1 and SMA in the PAI-1 null EC.

All of the works listed above suggested that TGF-*β* signal plays a critical role in cardiac fibrosis. It is of great interest whether EndMT-mediated cardiac fibrosis is regulated by other components of TGF-*β* signals and TGF-*β*-induced EMT-related transcription factors.

## 10. Roles of TGF-*β* Family Signals in the Cardiac Fibrosis That Does Not Involve EndMT

TGF-*β* is one of the critical regulators of cardiac fibrosis. Therefore, some groups have recently reported the roles of TGF-*β* family signals in cardiac fibrosis in which EndMT is not involved.

 GDF15 (also known as MIC-1), a secreted member of the TGF-*β* superfamily, was identified as an antihypertrophic regulatory factor in the heart [65]. GDF15 is not expressed in the normal adult heart but is induced in response to conditions that promote hypertrophy and dilated cardiomyopathy. GDF15 transgenic mice were normal but were partially resistant to pressure overload-induced hypertrophy. Conversely, GDF15 knockout mice showed enhanced cardiac hypertrophic growth following pressure overload stimulation. GDF15 knockout mice also demonstrated a pronounced loss in ventricular performance. GDF15 stimulation promoted phosphorylation of Smad2/3 in an in vitro analysis. Overexpression of Smad2 attenuated cardiomyocyte hypertrophy similar to GDF15 treatment, whereas overexpression of the inhibitory Smad proteins, Smad6/7, reversed the antihypertrophic effects of GDF15. Therefore, GDF15 is an autocrine/endocrine factor that antagonizes the hypertrophic response and loss of ventricular performance and may be a candidate molecule for treating cardiac hypertrophy clinically.

TGF-*β*-ALK-5 signals are involved in GATA-6-mediated angiogenic function and survival in ECs [66]. Knockdown of GATA-6 decreased proliferation, migration, and tube formation of ECs. This effect was partially rescued by TGF-*β*-neutralizing antibody or ALK-5 inhibitor. GATA-6 suppresses TGF-*β* expression in EC, which results in downregulation of phosphorylation of Smad2 without affecting phosphorylation of Smad1, suggesting that GATA-6 suppresses only TGF-*β*-ALK-5 signals but not TGF-*β*-ALK-1 signals for exerting angiogenic function. 

Sarcomere protein mutation (a-MHC R719W) causes hypertrophic cardiomyopathy, a disorder characterized by myocyte enlargement, fibrosis, and impaired ventricular relaxation. This mutation activates proliferative and profibrotic signals in nonmyocyte cells to produce TGF-*β*, and its responsive genes such as periostin. In treatment of TGF-*β*-neutralizing antibody or Losartan, an AT1 blocker abrogates nonmyocyte proliferation and fibrosis probably by blocking both TGF-*β* signals but through unknown precise mechanism [67]. Once again, TGF-*β* is one of the main causes of fibrosis here and can also be an efficient target for ameliorating fibrotic microenvironments in the heart.

## 11. Future Perspective and Concluding Remarks

The recent findings that EndMT is involved in different diseases suggest that modulating EndMT may serve a promising new therapeutic strategy for heart diseases and cancer. The endothelium is a promising target for drug delivery because it lies in direct contact with the bloodstream. Possible treatment strategies may target the TGF-*β* signaling pathways. We found that inhibition of endogenously activated TGF-*β* signals in ECs [68] by a small molecule that inhibits kinases of receptors for TGF-*β* led to a decrease in EndMT, suggesting that inhibition of TGF-*β* signals may suppress EndMT. Interestingly, Zeisberg and colleagues demonstrated that systemic administration of recombinant human BMP-7 (rhBMP-7) significantly reduced EndMT during cardiac fibrosis [1]. Additional studies are needed to identify the precise molecular mechanisms by which BMP-7 inhibits the EndMT. 

Furthermore, accumulating evidence suggests that EndMT not only induces the formation of fibroblasts in cardiac fibrosis but also in other fibrotic disorders including intestinal fibrosis [69] and kidney fibrosis [70]. In conclusion, EndMT is expected to be a target for the development of new therapies for multiple fibrotic disorders.
